# Multiscale Contrasts Between the Right and Left Ventricle Biomechanics in Healthy Adult Sheep and Translational Implications

**DOI:** 10.3389/fbioe.2022.857638

**Published:** 2022-04-21

**Authors:** Wenqiang Liu, Michael Nguyen-Truong, Kristen LeBar, Kevin M. Labus, Elisabeth Gray, Matt Ahern, Sunder Neelakantan, Reza Avazmohammadi, Kirk C. McGilvray, Christian M. Puttlitz, Zhijie Wang

**Affiliations:** ^1^ Cardiovascular Biomechanics Laboratory, School of Biomedical Engineering, Colorado State University, Fort Collins, CO, United States; ^2^ Cardiovascular Biomechanics Laboratory, Department of Mechanical Engineering, Colorado State University, Fort Collins, CO, United States; ^3^ Orthopaedic Bioengineering Research Laboratory, Department of Mechanical Engineering, Colorado State University, Fort Collins, CO, United States; ^4^ Computation Cardiovascular Bioengineering Lab, Department of Biomedical Engineering, Texas A&M University, College Station, TX, United States; ^5^ Computation Cardiovascular Bioengineering Lab, J. Mike Walker ’66 Department of Mechanical Engineering, Texas A&M University, College Station, TX, United States; ^6^ Department of Cardiovascular Sciences, Houston Methodist Academic Institute, Houston, TX, United States; ^7^ Orthopaedic Bioengineering Research Laboratory, School of Biomedical Engineering, Colorado State University, Fort Collins, CO, United States

**Keywords:** anisotropy, ovine, Fung exponential strain energy function, structurally informed model, collagen isoform

## Abstract

Cardiac biomechanics play a significant role in the progression of structural heart diseases (SHDs). SHDs alter baseline myocardial biomechanics leading to single or bi-ventricular dysfunction. But therapies for left ventricle (LV) failure patients do not always work well for right ventricle (RV) failure patients. This is partly because the basic knowledge of baseline contrasts between the RV and LV biomechanics remains elusive with limited discrepant findings. The aim of the study was to investigate the multiscale contrasts between LV and RV biomechanics in large animal species. We hypothesize that the adult healthy LV and RV have distinct passive anisotropic biomechanical properties. *Ex vivo* biaxial tests were performed in fresh sheep hearts. Histology and immunohistochemistry were performed to measure tissue collagen. The experimental data were then fitted to a Fung type model and a structurally informed model, separately. We found that the LV was stiffer in the longitudinal (outflow tract) than circumferential direction, whereas the RV showed the opposite anisotropic behavior. The anisotropic parameter *K* from the Fung type model accurately captured contrasting anisotropic behaviors in the LV and RV. When comparing the elasticity in the same direction, the LV was stiffer than the RV longitudinally and the RV was stiffer than the LV circumferentially, suggesting different filling patterns of these ventricles during diastole. Results from the structurally informed model suggest potentially stiffer collagen fibers in the LV than RV, demanding further investigation. Finally, type III collagen content was correlated with the low-strain elastic moduli in both ventricles. In summary, our findings provide fundamental biomechanical differences between the chambers. These results provide valuable insights for guiding cardiac tissue engineering and regenerative studies to implement chamber-specific matrix mechanics, which is particularly critical for identifying biomechanical mechanisms of diseases or mechanical regulation of therapeutic responses. In addition, our results serve as a benchmark for image-based inverse modeling technologies to non-invasively estimate myocardial properties in the RV and LV.

## 1 Introduction

In both the U.S. and worldwide, structural heart diseases (SHDs) are the leading cause of death. The progression of SHDs is associated with unique ventricular biomechanical alterations that affect either single or double sides of the ventricles. Diastolic dysfunction is common in many SHDs and confers poor outcome in both the left and right ventricular diseases including heart failure with preserved ejection fraction and pulmonary hypertension. Despite the development of modern therapies, effective treatments for diastolic dysfunction in heart failure patients remain limited. As diastolic dysfunction is directly influenced by the passive biomechanical behavior of the myocardium, a detailed knowledge of this behavior could facilitate developing new therapeutic targets and personalized treatment approaches for diastolic dysfunction. Although several studies have characterized the passive biomechanics of the myocardium (Avazmohammadi et al., 2019a), these studies often focus on either the left or right ventricle and the key question of how different the passive behavior of the two ventricles remains unanswered. This question becomes very important as often therapies that work for left ventricle (LV) failure patients (e.g., valsartan and pirfenidone) do not achieve similar effectiveness in the right ventricle (RV) failure patients (Andersen et al., 2019a; Pfau et al., 2019; SharifiKia et al., 2020), which indicates that different failing mechanisms, stemming from differences in baseline biomechanical behavior, exist between the ventricles and calls for the development of chamber-specific treatment.

Under physiological conditions, the LV and RV experience dramatically different hemodynamic environments: while the LV experiences a high pressure, high resistance and low compliance circulatory system, the RV experiences a low pressure, low resistance and high compliance circulatory system. The RV has been long considered as a “compliant” chamber compared to the LV because of its larger chamber compliance, an extrinsic mechanical property calculated by ΔV/ΔP over a cardiac cycle (i.e., the ratio of volume change to pressure change from end-diastole to end-systole). However, whether their intrinsic mechanical properties such as elastic modulus differ from each other remains a knowledge gap (VonkNoordegraaf et al., 2017; Andersen et al., 2019b). Moreover, in hypertensive remodeling, the RV can face as high as ∼5-fold increase of afterload under pulmonary hypertension, but the LV only faces a ∼1.5-fold increase of afterload in systemic hypertension (Abel, 1965; Andersen et al., 2019b; Pahuja and Burkhoff, 2019). Different baseline mechanics and mechanical afterloads may be responsible for the poor adaptation of the RV to pressure overload compared to the LV (GUYTON et al., 1954; LAVER et al., 1979). The RV also has been shown to have a higher collagen content (key contributor to ventricular biomechanics) than the LV (Oken and Boucek, 1957), which suggests that the extracellular matrix remodeling may be different for each chamber. Therefore, it is crucial to investigate the baseline biomechanical differences between the ventricles to further delineate the different mechanisms of and subsequent treatment for LV- vs. RV-associated SHDs.

To date, there are only a few studies directly comparing the mechanical properties of the LV and RV. Among earlier studies, Humphrey et al. reported the equibiaxial mechanical behavior of the canine epicardium tissues (Humphrey et al., 1990). The stress-stretch curves were similar between the LV and RV: the tissues were isotropic at low strains and became stiffer and anisotropic at high strains. But there was no further analysis of the elastic properties. Later, the canine RV biaxial properties were measured and compared with the literature LV data by Sacks and Chuong (1993).Their data, as well as the human myocardium data from Fatemifar et al., (2018), both suggested a stiffer RV compared to the LV in the main fiber direction. Javani et al. (2016) and Kakaletsis et al. (2021) measured adult ovine hearts by equibiaxial and multi-modal uniaxial tensile/compression and simple shear mechanical tests, respectively. These studies reported a stiffer material property of the LV than the RV in the main fiber direction. This finding is contradictory to the former studies. Recently, human ventricles were examined by Sommer et al. (2015) and the results suggested a stiffer RV than LV, although the sample size was small (*n* = 3 for RV). Since both human studies included samples from patients with and without cardiovascular diseases, the baseline information of healthy ventricles still remains unclear. Finally, healthy neonatal porcine hearts were examined by Ahmad et al. (2018) and the RV was shown to be stiffer than the LV in the main fiber direction in the developing myocardium. However, the ventricular wall is different in functionality and structure between the neonate and adult (Lindsey et al., 2005; Wang et al., 2013). Therefore, the mechanical difference between the RV and LV in healthy adults remains inconclusive.

The lack of baseline biomechanics data for LV and RV and particularly in large animal species has limited the expenditure of cardiac research in two main fields. Firstly, ventricular passive biomechanical properties can serve as a benchmark for image-based inverse modeling technologies to non-invasively estimate myocardial properties in the RV and LV (Mojsejenko et al., 2015; Yap et al., 2015; Avazmohammadi et al., 2019b; Avazmohammadi et al., 2019c). The image-based inverse model technology offers a promising platform to measure tissue-level stiffness and decouple fiber-level contributors to this stiffness. The information derived from this approach can significantly improve the diagnosis and prognosis that are solely based on global functional metrices (e.g., end-diastolic pressure-volume relation). Second, the design of biomaterials for cardiac tissue engineering and regenerative medicine encompasses a wide range of substrate elasticity from sub- to supra- physiological stiffnesses (20 kPa–92 MPa), as we recently reviewed (Nguyen-Truong et al., 2020a). There are two layers of problems here. The biomaterial stiffnesses are oftentimes outside of the physiological range of myocardium stiffness, and most studies used the benchmark of LV stiffness, which leads to the lack of RV-specific therapy development (Nguyen-Truong et al., 2020a) in this rapidly expanding research field.

With the persistent need to establish computational tools for the estimation of large animal (including human) cardiac biomechanics and to guide cellular and tissue bioengineering research, the goal of this study is to compare the biomechanical properties of the LV and RV in healthy adults. We hypothesize that the adult healthy LV and RV have distinct passive anisotropic biomechanical properties. Sheep were chosen for their closer similarities to adult human anatomy, function, and physiology (Camacho et al., 2016) than small animals, and thus the findings are more translatable to human cardiac biomechanics. We measured the passive biaxial properties of ovine LV and RV and quantified collagen distribution in the tissues. Moreover, there is increasing agreement that a constitutive model that incorporates the microstructural information has greater potential to characterize the heterogenous mechanical behavior of myocardium (Avazmohammadi et al., 2017a; Avazmohammadi et al., 2018). Equibiaxial data were then fitted to a four-parameter Fung type model (Matsumoto et al., 2009; Javani et al., 2016) and a structurally informed model (Avazmohammadi et al., 2017a) with additional measurements from serial histology sections on the myo-/collagen fibers, separately. Our results indicated significant discrepancies between anisotropic behaviors of the LV and RV (relative to OT coordinates) that were concisely described by the anisotropic parameter *K* derived from the Fung type model. The structurally informed model indicated stiffer collagen fibers in the LV than the RV, which awaits further investigation. Furthermore, the elasticity at low strains was correlated with type III collagen content in both ventricles. These findings advance the fundamental understanding of the differences between LV and RV biomechanics, which can be used to guide cardiac tissue engineering and regenerative studies with chamber-specific mechanical environments and to develop the image-based inverse modeling technologies to non-invasively estimate myocardial properties in the ventricles.

## 2 Materials and Methods

### 2.1 Tissue Sample Preparation

**FIGURE 1:**
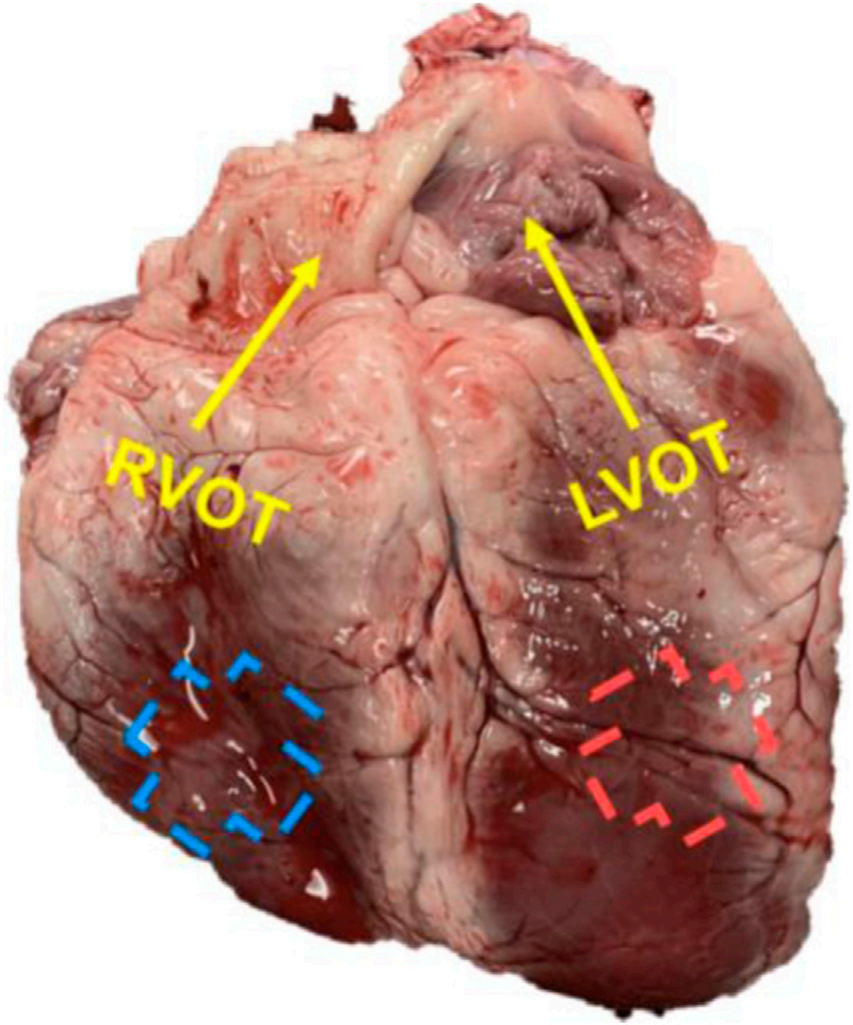
A representative image of the ovine heart with the labeling of the outflow (OT, longitudinal) direction for each ventricle.

Fresh hearts (*n* = 11) were obtained from 4+ year-old female sheep with no known cardiovascular disease or defects after the animals were euthanized for unrelated studies. Within 4 h of sacrifice, the tissues were immersed in physiological saline solution (PBS) at room temperature until mechanical testing (Witzenburg et al., 2012). The outflow tract (OT) direction was used as the longitudinal direction as in previous studies (Valdez-Jasso et al., 2012; Jang et al., 2017; Liu et al., 2021; Hill et al., 2014). A cruciform section (total dimensions: 30 mm × 30 mm; center square dimensions: 20 mm × 20 mm) was cut from each ventricle in a similar anatomic region (anterior free wall, with similar distance to the apex and base) and free of fibrotic deposition (Figure 1). For both ventricles, we used the middle layer for mechanical tests after cleaning of the endocardial and epicardial surfaces, including the removal of papillary muscles and trabeculae. The tissue thickness was ∼3–4 mm in all testing samples to achieve negligible shear deformation requirement for the biaxial test (Labus and Puttlitz, 2016).

### 2.2 Biaxial Testing

The sample was then mounted onto an in-house biaxial tester and then *ex vivo* mechanical tests were performed at room temperature, with a regular spray of PBS solution to keep the tissue moist. Prior to testing, graphite powder (AGS, MI) was dusted onto the sample for strain characterization. Before testing, approximately 0.1 N of force was applied to pre-load the tissue in both directions. Next, biaxial testing was performed at two different displacement ratios (longitudinal:circumferential) in random order (2:1 and 1:2) and then at an equibiaxial test (2:2). The first ratio test was completed with 15 cycles including preconditioning cycles. The following ratios’ tests were completed with eight cycles per ratio. Finally, the first ratio test was repeated to confirm that no tissue damage occurred. Data acquisition was performed with an in-house LabVIEW program (Labus and Puttlitz, 2016).

Each sample underwent a maximum of 25% strain following the reported physiological strains (Rappaport et al., 2006) and the maximum strain rate was 1% s^−1^. Sample images were taken with a CCD camera (Nikon) at one fps and tissue deformations were obtained by digital image correlation (Labus and Puttlitz, 2016). The digital image correlation was applied to the region of interest (ROI), which was the central, square-shaped region far enough from the boundaries, and we have verified that the deformation in the ROI was nearly homogeneous. The Cauchy (σ) and the second Piola-Kirchhoff (P-K) (**S**) stresses, and Green strain (**E**) were calculated for each direction ( $\sigma =\lambda_{i}P$ , $S=P/\lambda_{i}$ , and $P=F/A_{0}$ , $E=\frac{1}{2}\left(\lambda_{i}^{2}-1\right)$ , where F is the measured force, P is the engineering stress, A_0_ is the initial cross-section area, and $\lambda_{i}$ is the stretch in the i=L, C direction with L and C subscripts denoting the longitudinal and circumferential directions, respectively), where the width and thickness were the original dimensions at no load. The elastic moduli (*M*) were derived as the slopes of the stress-strain curves at the low and high strain ranges (i.e., the first and last 20% of the loading stress-strain curve) (Jang et al., 2017). Then, the ratio *M*/ε in the low or high strain range was used to assess the modulus (*M*) normalized by the maximal Green strain (ε) in the corresponding strain range and the respective direction (Liu et al., 2021).

### 2.3 Constitutive Modeling

Next, we fitted our equibiaxial experimental data to a phenomenological constitutive model (Fung type model) and a structurally informed model. We used the phenomenological Fung type model to assess the overall *tissue-level* stiffness and anisotropic behavior in the LV and RV in a comparative manner; and the structurally informed model was used to provide insights into the differences between *fiber-level* mechanical and architectural properties of the LV and RV myocardium. An affine deformation was assumed within the samples under equibiaxial loading as commonly adopted in biaxial soft tissue testing.

#### 2.3.1 Fung Type Model

We calculated the shear deformations *k*_*LC*_ and *k*_*CL*_ from our experimental data. (Table 1 **)**. The shear deformation was minimal compared to the in-plane stretches along longitudinal (OT) and circumferential (cross-OT) directions. Accordingly, shear stresses were assumed to be negligible in our modeling work.

**TABLE 1:** Maximum stretches in the in-plane and shear directions during equibiaxial tests. Data are presented as mean ± SEM.

Ventricle	*λ*_*L*_	*λ*_*C*_	*k*_*LC*_ (shear)	*k*_*CL*_ (shear)
LV (*n* = 7)	1.14±0.02	1.19±0.02	1.04±0.01 ^a^ ^,^ ^b^	1.02±0.02 ^a^ ^,^ ^b^
RV (*n* = 7)	1.20±0.02	1.14±0.01	1.02±0.01 ^a^ ^,^ ^b^	1.03±0.01 ^a^ ^,^ ^b^

a *p* < 0.001 vs. *λ*_*L*_.

b *p* < 0.001 vs. *λ*_*C*_.

*λ*_*L*_, stretch in the longitudinal direction; *λ*_*C*_, stretch in the circumferential direction; *k*_*LC*_ and *k*_*CL*_, stretches in the shear directions.

Furthermore, the relevant Green strain tensor (**E**) components were calculated as:

$$E_{L}=\frac{1}{2}\left(\lambda_{L}^{2}-1\right),E_{C}=\frac{1}{2}\left(\lambda_{C}^{2}-1\right)$$ (1)

where $\lambda_{L}$ and $\lambda_{C}$ are the stretch in longitudinal and circumferential directions, respectively.

Next, a four-parameter Fung type constitutive model (Matsumoto et al., 2009; Javani et al., 2016) with the following energy function (Ψ) was fit to the biaxial stress-strain data:

$$\text{Ψ}=\frac{B}{2}\left(e^{Q}-1\right),Q=b_{L}E_{L}^{2}+2b_{LC}E_{L}E_{C}+b_{C}E_{C}^{2}$$ (2)

The second P-K and Cauchy stresses for an incompressible tissue were calculated as:

$$S=2\frac{\partial \text{Ψ}}{\partial C}-pC^{-1},\sigma =\mathrm{FS}F^{T}$$ (3)

where **F** is the deformation gradient tensor, **C** is the right Cauchy-Green tensor, *p* is an unknown hydrostatic pressure to enforce det(**C**) = 1, and $b_{L}$ , $b_{LC}$ , $b_{C}$ and *B* are the material constants. The stress-strain relationships in the L and C directions were derived as:

$$\sigma_{L}=\left(2E_{L}+1\right)\left(b_{L}E_{L}+b_{LC}E_{C}\right)Be^{\left(b_{L}E_{L}^{2}+2b_{LC}E_{L}E_{C}+b_{C}E_{C}^{2}\right)}$$

$$\sigma_{C}=\left(2E_{C}+1\right)\left(b_{LC}E_{L}+b_{C}E_{C}\right)Be^{\left(b_{L}E_{L}^{2}+2b_{LC}E_{L}E_{C}+b_{C}E_{C}^{2}\right)}$$ (4)

The Fung strain energy function was fitted to the equibiaxial experimental data for each sample. The fitting was performed in MATLAB. The sensitivity to initial guesses was checked for every fit and a minimal dependency to initial guess was found for all the fits. The root mean square (RMS) was calculated to assess the fitting results. Finally, the anisotropic parameter *K* and elasticity at zero load in two directions ( $M_{0,L}$ and $M_{0,C}$ ) were calculated as described in previous studies (Matsumoto et al., 2009; Javani et al., 2016; Nguyen-truong et al., 2021).

#### 2.3.2 Structurally Informed Model

Next, we used a structurally informed model that incorporates the transmural changes of myo-/collagen fibers to reveal the contributions of each fiber type to the tissue-level myocardial biomechanical behavior (Fan and Sacks, 2014; Avazmohammadi et al., 2017a). Briefly, the total energy function (Ψ) was written as the sum of the mechanical contributions of the ground matrix and myo- and collagen fibers as,

$$\text{Ψ}\left(C\right)=\phi^{g}{\text{Ψ}}^{g}\left(C\right)+\phi^{m}{\text{Ψ}}^{m}\left(C\right)+\phi^{c}{\text{Ψ}}^{c}\left(C\right)$$ (5)

where $\Phi^{g},\Phi^{m}\;\text{and}\;\Phi^{c}$ are volume fractions for the ground matrix (including non-structural extracellular matrix proteins, fibroblasts, interstitial fluid, etc.), myo- and collagen fibers, respectively, and $\;{\text{Ψ}}^{g},{\text{Ψ}}^{m},\;\text{and}\;{\text{Ψ}}^{c}$ are strain energy functions associated with each phase. The volume fraction measurement is described in the next section (see §2.4.2).

At the tissue level, the 2nd P-K stress tensor (**S**) was described in terms of the energy function Ψ(C) :

$$S=2\frac{\partial \text{Ψ}}{\partial C}-pC^{-1}=S^{g}+S^{m}+S^{c}$$ (6)

The stress-strain relationships for ground matrix, myo- and collagen fibers were derived as:

$$S^{g}=\phi^{g}k^{g}I-pC^{-1}$$ (7)

$$S^{m}=\frac{\phi^{m}k_{1}^{m}}{H}\overset{H}{\underset{0}{\int }}\overset{\pi /2}{\underset{-\pi /2}{\int }}{\text{Γ}}^{m}\left({\text{θ}}^{m},z\right)\frac{\left(\sqrt{I^{m}}-1\right)}{\sqrt{I^{m}}}\times \exp\left[k_{2}^{m}{\left(\sqrt{I^{m}}-1\right)}^{2}\right]\left(n^{m}⊗n^{m}\right)d{\text{θ}}^{m}dz$$ (8)

$$S^{C}=\left\{ \begin{array}{l} \frac{\phi^{c}k_{1}^{c}}{H}\overset{H}{\underset{0}{\int }}\overset{\pi /2}{\underset{-\pi /2}{\int }}{\text{Γ}}^{c}\left({\text{θ}}^{c},z\right)\times \left(e^{k_{2}^{c}\;E^{c}}-1\right)\left(n^{c}⊗n^{c}\right)d{\text{θ}}^{m}dz,\;for\;E^{c}\leq E_{ub}\\ \frac{\phi^{c}k_{1}^{c}}{H}\overset{H}{\underset{0}{\int }}\overset{\pi /2}{\underset{-\pi /2}{\int }}{\text{Γ}}^{c}\left({\text{θ}}^{c},z\right)\left[\left(e^{k_{2}^{c}E^{c}}-1\right)+k_{2}^{c}\;e^{k_{2}^{c}\;E^{c}}\left(E^{c}-E_{ub}\right)\right]\left(n^{c}⊗n^{c}\right)d{\text{θ}}^{m}dz,\;for\;E^{c}>E_{ub} \end{array}\right.$$ (9)

where **I** is the identity tensor, $I^{m}=2\left(n^{m}\cdot En^{m}\right)+1,E^{c}=n^{c}\cdot En^{c}\;$ are the pertinent kinematic measures, *k*’s are model parameters, and **n**’s are the defined unit vectors that describe the planar orientations of the myo-and collagen fibers, respectively. We adapted the previous model and derived the fiber orientation parameters ${\text{θ}}^{m}\;and\;{\text{θ}}^{c}$ (ranged from 0 to π/2 ) from serial histology measurements (see §2.4.2). The parameters describing the transmural orientation distribution (Γ) were derived for myo- and collagen fibers using a modified Beta distribution function (Avazmohammadi et al., 2017a). In the transmural direction, the normalized tissue thickness was denoted as H and a value of 100 represents the entire tissue thickness. *E*_*ub*_ is the upper bound of the transition region derived from the average stress-strain curve calculated from each direction.

The myofiber model parameter $k_{1}^{m}$ was estimated from the fit to experimental data in the low strain region as described previously (Hill et al., 2014), and the parameter for amorphous ground matrix $k^{g}$ was fixed at a value of 10 kPa due to its much lower contribution to the mechanical behavior of myocardium compared to that of myo-/collagen fibers (Humphrey and Jay, 2002). The same fitting method was used as described in the above Fung type model section.

### 2.4 Microstructural Measurements

After biaxial testing, the samples were fixed in 10% buffered formalin and embedded in paraffin for collagen content measurement. In some samples (*n* = 3 for LV and *n* = 4 for RV), the tissue blocks were further sectioned into 4–8 serial sections (∼125 µM apart) from the epicardial to endocardial side and stained for fiber orientation measurement.

#### 2.4.1 Collagen Content Measurement

The tissue slices were stained with Picrosirius Red (PSR) and imaged and analyzed *via* a transmission microscope (Nikon Eclipse E800) and Image Pro Premier software (Media Cybernetics, Rockville, MD) for collagen content quantification. For each sample, three regions were randomly selected under polarized light microscopy. An image thresholding method in which yellow, green, brown and dark blue colors were chosen to represent type I collagen, type III collagen, ground matrix and muscle, respectively. The amounts of type I and III collagen were quantified as the area percentage to total tissue area, and the amount of collagen content was quantified as the total area percentage of type I and III collagen (Namba et al., 1997; Nguyen-Truong et al., 2020b).

We further performed immunohistochemistry (IHC) to LV (*n* = 4) and RV (*n* = 4) samples to confirm the area fraction measurement of type III collagen in PSR staining slides. Tissue samples were stained with rabbit polyclonal anti-human collagen III antibody (1:500 dilution, ab7778, Abcam, Cambridge, United Kingdom). Image thresholding via ImageJ (U.S. NIH, Bethesda, Maryland) was used to determine the area fraction of collagen III.

#### 2.4.2 Myo- and Collagen Fibers Orientation Measurement

From the serial histology sections with PSR staining, the transmural change of fiber orientation was measured using in-house MATLAB codes adapted from a previous study (Avazmohammadi et al., 2017a). Briefly, each serial tissue slice was imaged under bright field light microscopy and separated by the color of tissue component using ColorDeconvolution2, a plug-in in ImageJ. Fiber angle was calculated for collagen and myofiber, respectively. The transmural fiber orientation distribution was then represented by a Beta distribution function to fit a surface to the 3D data. The mean transmural fiber orientations for both myo- and collagen fibers were calculated from the Hermitian fit parameters (Avazmohammadi et al., 2017a). These data were used to derive the transmural orientation distribution (Γ) for the structurally informed modeling.

### 2.5 Statistical Analysis

Comparisons between directions (longitudinal versus circumferential) were performed with the Wilcoxon signed-rank test for the paired equibiaxial data. For all other statistical comparisons, the Mann-Whitney U test was used for the unpaired data. Pearson correlation analysis was performed to investigate the correlations between the modeling parameters or collagen content and mechanical properties. All analyses were performed in GraphPad Prism (v8.0.2). Data are presented as mean ± SEM and *p* < 0.05 was considered statistically significant.

## 3 Results

### 3.1 Differences in Elastic Behaviors Along Each Direction

**FIGURE 2:**
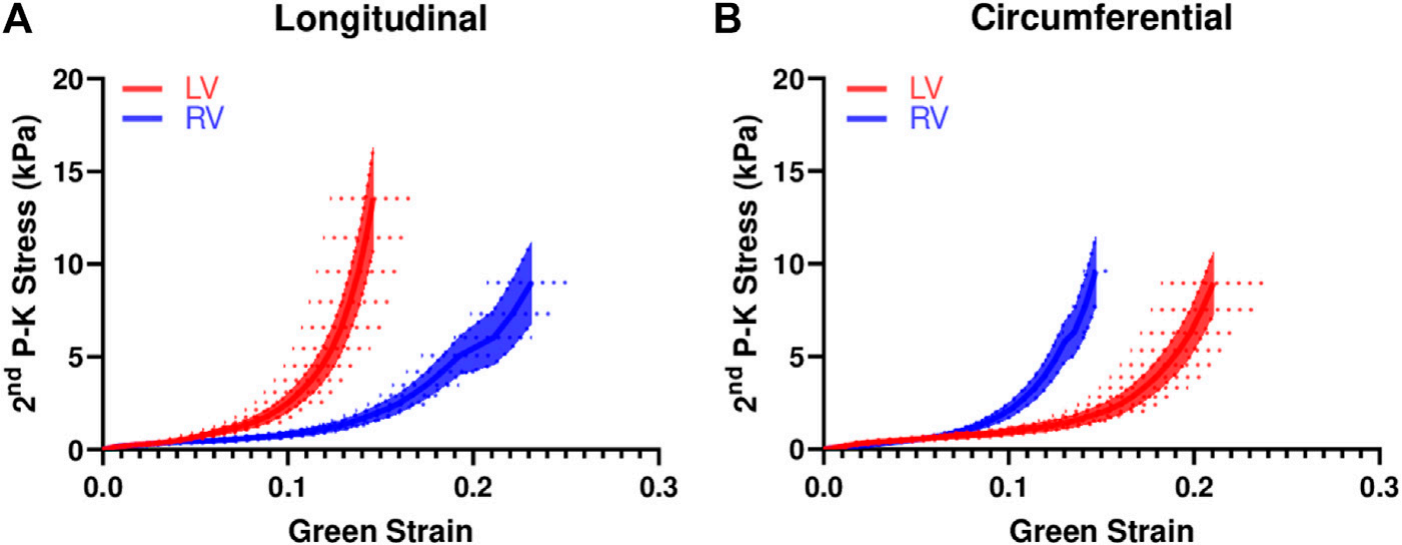
Average equibiaxial stress-strain curves in the longitudinal **(A)** and circumferential **(B)** directions in the LV and RV (*n* = 7 per group). The shaded area is the standard error of the stress data, and the dash line is the standard error for the strain data.

The average stress-strain curves from equibiaxial tests are shown in Figure 2. The curves for the LV were leftward of the curves for the RV in the longitudinal direction (Figure 2A), indicating a stiffer mechanical property of the LV in this direction. The opposite behavior was observed in the circumferential direction (Figure 2B), indicating a stiffer mechanical property of the RV in this direction. Similar behavior was observed from the stress-strain data from non-equibiaxial tests.

**FIGURE 3:**
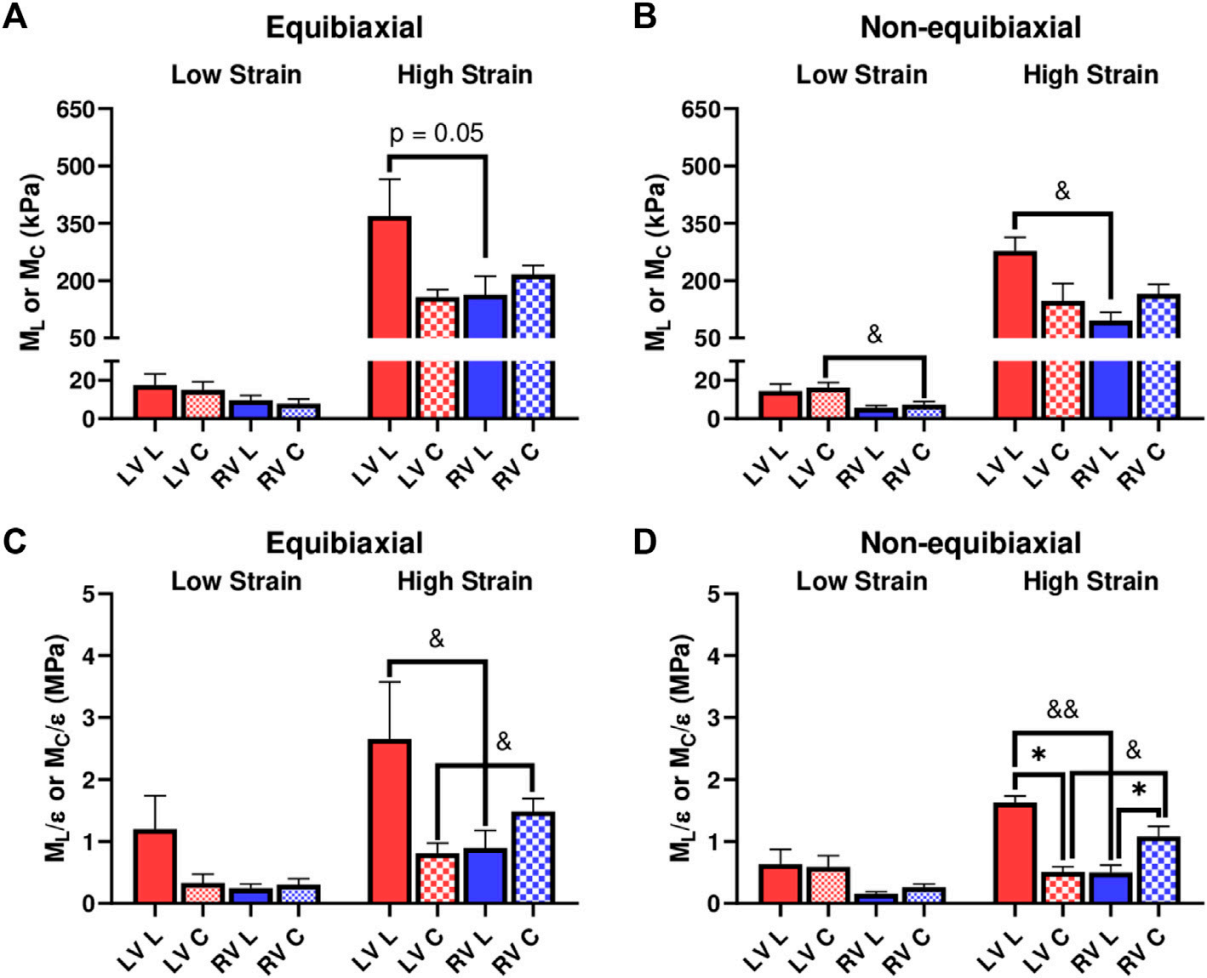
**(A,B)** Elastic moduli (*M*) of LVs and RVs from the equibiaxial **(A)** and non-equibiaxial tests **(B)**. **(C,D)** Strain-weighted elastic moduli (*M*/ε) of LVs and RVs from the equibiaxial **(C)** and non-equibiaxial tests **(D)**. For the equibiaxial tests, *n* = 7 per group; for the non-equibiaxial tests, LV, L: *n* = 4; LV, **(C)** *n* = 5; RV, L: *n* = 7; RV, **(C)** *n* = 6. *: *p* < 0.05 comparison between the directions and *p* < 0.05 and *p* < 0.01 for comparison between the ventricles.

We further investigated the elastic moduli at low and high strain ranges, which typically represent the mechanical behavior of myofibers and collagen fibers, respectively. At the low strains, the ventricles presented similar properties except that the LV showed a higher circumferential *M* than the RV for the non-equibiaxial tests (Figure 3B, *p* = 0.052). At the high strains, the LV had a higher *M* or *M*/ε than the RV in the longitudinal direction, whereas the RV had a higher *M* or *M*/ε than the LV in the circumferential direction (Figure 3).

### 3.2 Differences in Anisotropic Behaviors

We compared the *M* and *M*/ε between the two axes to examine the anisotropic behavior of the tissue. For the LV, the *M* and *M*/ε in the circumferential direction were significantly smaller than those in the longitudinal direction (Figure 3D), indicating that the LV was stiffer in the longitudinal direction. In contrast for the RV, the *M* and *M*/ε in the circumferential direction were higher compared to the longitudinal direction (Figure 3D), suggesting that the RV was stiffer in the circumferential direction. Therefore, the LV and RV had different anisotropic behaviors.

### 3.3 Experimental Data Fitting With Fung Type Model

**FIGURE 4:**
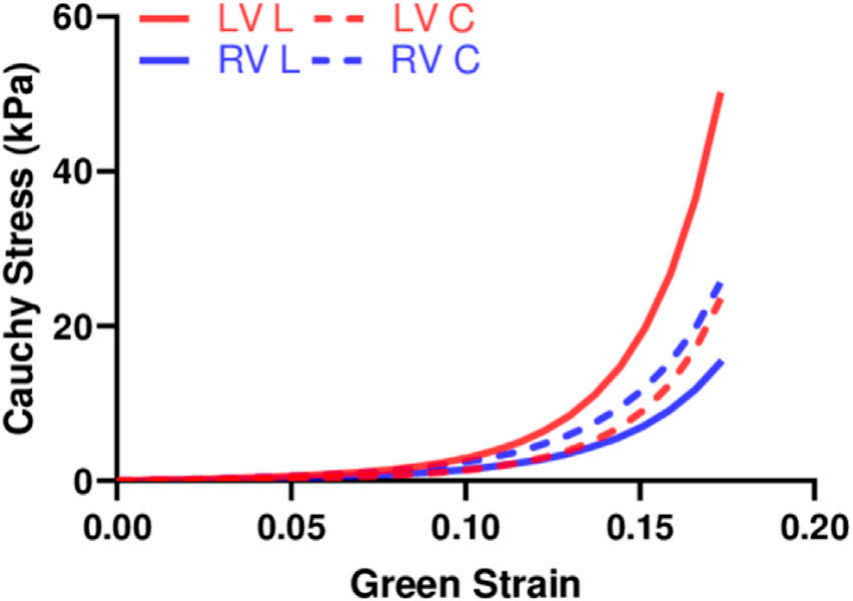
Simulated equibiaxial stress-strain curves generated by the four-parameter Fung model, using the average values of the fitting parameters for both ventricles in two directions.

We performed fitting of the equibiaxial stress-strain curves using the four-parameter Fung type model. A good fit to the experimental data was observed for both ventricles and at both directions (low RMS values), and the fitting results are summarized in Table 2. The simulated equibiaxial stress-strain curves using the mean values of the estimated constants showed similar behaviors as our experimental data: the LV and RV had different anisotropic behaviors (Figure 4) and the LV was stiffer than the RV in the longitudinal direction (Figure 4).

**TABLE 2:** Fung model fitting results. Average data are presented as mean ± SEM.

Ventricle	*b*_*L*_	*b*_*C*_	*b*_*LC*_	*B* (kPa)	RMS (kPa)
LV #1	145.67	25.16	$4.40\times 10^{-11}$	0.15	0.63
LV #2	41.63	17.91	0.01	0.09	0.09
LV #3	103.43	13.94	0.01	0.18	0.58
LV #4	96.74	53.68	0.01	0.17	0.14
LV #5	42.00	83.08	$1.33\times 10^{-10}$	0.10	0.28
LV #6	38.36	37.54	0.01	0.03	0.60
LV #7	33.51	27.33	0.01	0.88	0.25
LV (*n* = 7)	71.62±16.52	36.95±9.17	0.007±0.002	0.23±0.11	0.37±0.09
RV #1	17.44	35.07	$8.48\times 10^{-14}$	0.23	0.25
RV #2	45.49	20.05	0.01	0.38	0.24
RV #3	20.96	46.14	0.01	0.25	0.22
RV #4	57.19	79.55	$1.55\times 10^{-10}$	0.13	0.19
RV #5	51.30	99.99	0.01	0.07	0.42
RV #6	28.42	43.74	$8.29\times 10^{-11}$	0.15	0.10
RV #7	20.95	48.57	0.01	0.07	0.17
RV (*n* = 7)	34.54±6.20	53.30±10.32	0.006±0.002	0.18±0.04	0.23±0.04

$b_{L},b_{C},b_{LC}$ and *B* are the material constants, and RMS is root mean square.

**FIGURE 5:**
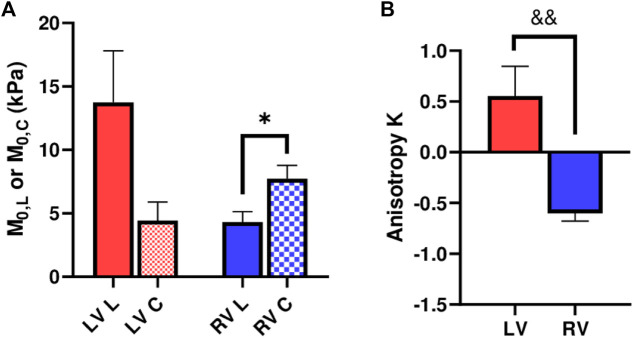
**(A)** Longitudinal and circumferential zero-load elastic modulus *M*_*0*_ for each ventricle type, and **(B)** Anisotropic parameter *K* for each ventricle type. *: *p* < 0.05 comparison between the directions and *p* < 0.01 comparison between the ventricles, respectively.

We further compared the zero-load modulus $M_{0}$ in each ventricle and in each direction, using the model fitting parameters. The $M_{0,C}\;$ was significantly larger than the $M_{0,L}$ in the RV (p < 0.05), and there was a strong trend of larger $M_{0,L}$ compared to the $M_{0,C}$ in the LV, indicating different anisotropic behaviors between the ventricles (Figure 5A **)**. Expectedly, the anisotropic parameter *K* was significantly different between the LV and RV (Figure 5B, *p* < 0.01). Finally, we performed correlation analyses and found that *K* was significantly correlated with the ratios of longitudinal to circumferential elastic moduli (*M* and *M*/ε) in both strain ranges (*p* < 0.05, r = 0.75–0.85, data not shown).

### 3.4 Experimental Fitting With Structurally Informed Model

**FIGURE 6:**
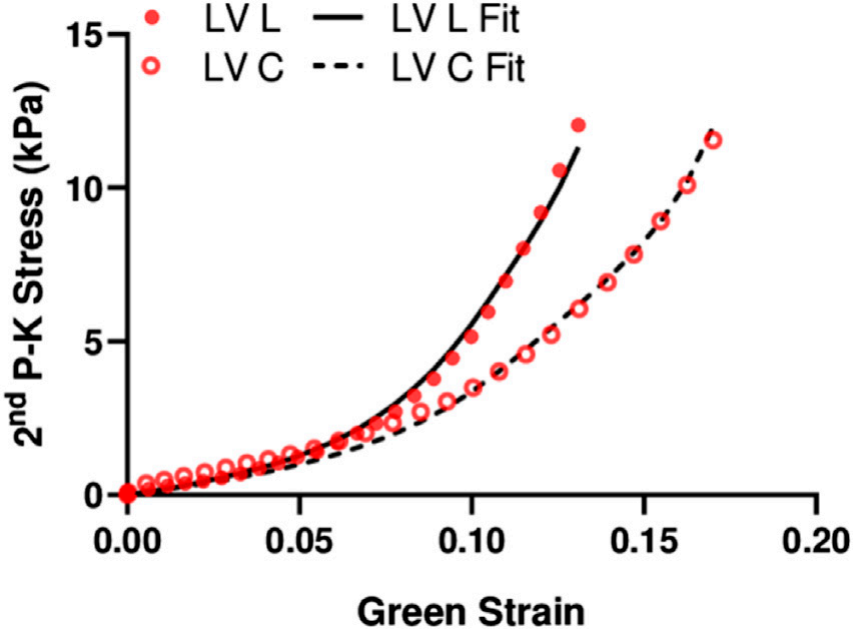
Representative fitting results using the structurally informed model.

We fit the equibiaxial stress-strain curves with the structurally informed model to investigate the different contributions of myocardial components (myo- and collagen fibers) to tissue mechanics in these chambers. The fitting results are summarized in Table 3, and a representative fitting result is shown in Figure 6. The model fit our experimental data well, which is evident by the small values of RMS. Compared to the RV, the LV tended to have larger stiffness for myo- and collagen fibers (see $k_{1}^{m}$ and $k_{1}^{c}$ ). Furthermore, the LV tended to have a larger transition strain (*E*_*ub*_) than that of the RV.

**TABLE 3:** Structurally informed model fitting results. Average data are presented as mean ± SEM.

	Myofiber		Collagen		RMS	
	$k_{1}^{m}$ (kPa)	$k_{2}^{m}$	$k_{1}^{c}$ (MPa)	$k_{2}^{c}$	*E*_*ub*_	(kPa)
LV #1	5.04	384.38	21.61	56.70	0.14	0.25
LV #2	15.77	301.81	69.41	38.15	0.2	0.65
LV #3	54.65	304.28	12.52	26.35	0.10	0.27
LV (*n* = 3)	25.15 ± 15.07	330.16 ± 27.12	34.51 ± 17.64	40.40 ± 8.83	0.15 ± 0.03	0.39 ± 0.13
RV #1	25.10	10.07	9.73	34.54	0.14	0.87
RV #2	9.87	$1.54\times 10^{-9}$	10.08	68.37	0.09	0.37
RV #3	26.55	573.59	12.68	59.94	0.13	1.28
RV #4	18.05	263.93	28.63	25.34	0.14	0.68
RV (*n* = 4)	19.89 ± 3.82	211.89 ± 135.14	15.28 ± 4.50	47.05 ± 10.20	0.12 ± 0.01	0.80 ± 0.19

*k*’s are model parameters, *E*_*ub*_ is the upper bond of the transition region.

### 3.5 Collagen Content Correlated With Low-Strain or High-Strain Elasticity in all Ventricles

**FIGURE 7:**
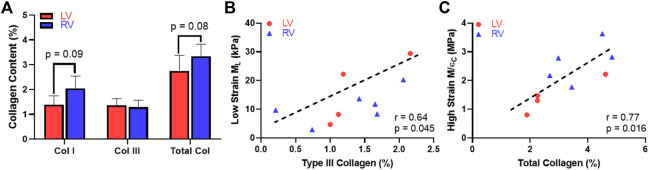
Histological measurement and correlation analysis for the collagen. **(A)** Variations in the content of collagen isoforms and the total collagen between the LV and RV, **(B)** Significant correlation between the type III collagen content and longitudinal *M* (*M*_L_) at the low-strain range and **(C)** Significant correlation between the total collagen content and circumferential *M/ε* at the high-strain range.

Next, we examined the difference in collagen content between the LV and RV. There were trends of higher total collagen and type I collagen contents in the RV compared to the LV (Figure 7A, *p* < 0.1). Our immunohistochemistry measurement of collagen III isoform agreed with the polarized light PSR measurement in collagen III. Furthermore, we observed a significant correlation between type III collagen percentage and the longitudinal *M* (*M*_L_) at the low-strain range in all samples (Figure 7B, *p* < 0.05). In addition, we observed a significant correlation between the total collagen and circumferential *M*/ε at the high-strain range (Figure 7C, *p* < 0.05). The complete results of all correlations between the collagen content and mechanical properties of the LV and RV are summarized in Table 4.

**TABLE 4:** Summary of all correlation results between the collagen content and the mechanical properties of all LV and RV samples.

	M _Low_L_	M/ε _Low_L_	M _High_L_	M/ε _High_L_	M _Low_C_	M/ε _Low_C_	M _High_C_	M/ε _High_C_
Col I	NS	NS	NS	NS	NS	NS	NS	*p* = 0.07
Col III	*	NS	NS	NS	NS	NS	NS	NS
Col T	NS	NS	NS	NS	NS	NS	NS	*

^*^ *p* <0.05.

Col I, Col III and Col T are the collagen type I, III and total collagen, respectively. L and C are longitudinal and circumferential directions, respectively.

NS, no significance.

## 4 Discussion

In this study, we aimed to compare the passive biaxial mechanical properties of the LV and RV in healthy adult ovine. We found that (Avazmohammadi et al., 2019a) the LV and RV had different anisotropic behaviors, with the LV being stiffer in the longitudinal (OT) than circumferential (cross-OT) direction and the RV showing the opposite result; (SharifiKia et al., 2020) the RV was more compliant than the LV in the longitudinal direction, and was stiffer than the LV in the circumferential direction; (Andersen et al., 2019a) the anisotropic parameter *K* derived from the Fung type model provided consistent finding in the opposite anisotropy of LV and RV as observed experimentally; (Pfau et al., 2019) using the structurally informed model, the LV was predicted to have stiffer collagen fibers than the RV (VonkNoordegraaf et al., 2017) the type III collagen played an important role in the longitudinal elasticity in all ventricles, especially at the low strain range. These findings provide fundamental information on the biomechanics of the LV and RV, which is valuable for the design of tissue and regenerative engineering studies and the development of image-based inverse modeling technologies to non-invasively estimate myocardial properties.

### 4.1 Different Anisotropic Behaviors Between the Left Ventricle and Right Ventricle

Both the LV and RV are reported to present anisotropic behaviors in prior studies, but the anisotropic behavior is inconsistent even within the same animal species. For instance, studies have found the healthy rat RV is stiffer in the longitudinal compared to circumferential direction (Valdez-Jasso et al., 2012; Hill et al., 2014). These findings are supported by longitudinal stress-strain curves being shifted leftward relative to the circumferential stress-strain curves in rodent RV (Park et al., 2016). However, another study reporting the low and high strain *M*’s suggests that the rat RV was stiffer in the circumferential compared to longitudinal direction, and the difference reversed and became larger in pulmonary hypertensive animals (Jang et al., 2017). These studies used the same (anatomical) coordinate system as in the present study, yet the anisotropic behavior of the RV still remains inconclusive. Moreover, it is unclear if the small and large animal species share similar anisotropic behaviors in the myocardium, and thus it is imperative to investigate the biaxial properties in large animal species independently.

In this study, we found that the ovine LV and RV had different anisotropic behaviors: the LV had larger *M* in the longitudinal direction compared to the circumferential direction, whereas the RV had the opposite trend of difference between these directions. The anisotropy parameter *K* derived from constitutive modeling was significantly correlated with the experimental data, confirming the different anisotropic behaviors. We speculate that the reason for this difference is the different need to facilitate blood filling and ejection in each ventricle. The LV is more conically shaped and is comprised of helical fibers that allows it to deform more circumferentially during cardiac cycles (majority of LV contractility occurs due to circumferential shortening); in contrast, the RV is crescent shaped and is comprised of wrap-around transverse fibers that deforms more longitudinally (majority of RV contractility occurs due to longitudinal shortening) (Friedberg and Redington, 2014; Prisco et al., 2020). Thus, our findings show a more compliant passive mechanical property of the wall in the main axis of cyclic deformation for both ventricles, which suggests that passive elasticity is maintained at a low level to reduce the elastic “resistance” and facilitate blood filling (as well as “pumping” potentially). The understanding of the differences in baseline anisotropy between the two ventricles will help to develop chamber-specific therapies aimed at reducing wall stress along the main axis of deformation.

### 4.2 Different Elasticity at Each Axis Between the Left Ventricle and Right Ventricle

To date, discrepant findings are reported on the comparison of elasticity of the LV and RV. Please note that the intrinsic mechanical property measurements should be distinguished from the general “consensus” that the RV is a more compliant chamber (an extrinsic mechanical measurement). In human myocardium, the RV tissue tended to achieve higher wall stresses in both biaxial axes compared to the LV tissue, but whether the difference reached statistical significance is unknown (Sommer et al., 2015). In contrast, various mechanical tests (biaxial test, triaxial shear test, uniaxial tensile/compression tests) on healthy ovine hearts showed that the LV was “overall” stiffer compared to the RV (Javani et al., 2016; Kakaletsis et al., 2021). Another recent study characterized neonatal porcine ventricles and found no difference between LV and RV stress-strain curves nor peak engineering stress (Ahmad et al., 2018). Hence, although all these studies used a different definition of biaxial axes (the main fiber and cross-fiber coordinate system), it remains unclear if the LV and RV have distinct intrinsic elastic property and how different they are.

The present study is the first investigation on the biaxial behavior of the ventricles in large animal species using an anatomical coordinate system (more adopted in the RV research area). Our results showed that the RV was stiffer than the LV in the circumferential direction, and the opposite trend of difference (i.e., LV was stiffer than the RV) was shown in the longitudinal direction (Figures 2, 3 **)**. Because of the different trends of comparison at different directions, it is not appropriate to simply conclude that the “LV is stiffer than the RV” or vice versa at the tissue level. Moreover, the heterogenous organization of myofiber layers and the nonlinear, anisotropic nature determine that one cannot refer to a single value of mechanical parameter (e.g., elastic modulus) to describe the myocardium. Unfortunately, such knowledge has not been well recognized by emerging fields like cell and tissue engineering for cardiac research. There are also mixed citations of “tensile” and “compressive” elastic modulus to represent the stiffness of myocardium. As a result, a variety of elastic moduli (from ones of kPa to tens of MPa) has been adopted for the matrix or bioscaffold to simulate ventricles in the tissue engineering or mechanobiology studies (Reis et al., 2016; Nguyen-Truong et al., 2020b). Lastly, there is no distinction between the LV and RV tissue mechanics due to the lack of knowledge of baseline contrasts of their biomechanical properties. Our data provides fundamental information on the LV versus RV passive, anisotropic mechanical behaviors. The data collected on large animal species further offers valuable data for translational applications in exploring mechanically regulated disease mechanism and/or regenerative therapy. We highly recommend future studies to incorporate the anisotropic, nonlinear elastic behavior of myocardium into considerations to better mimic *in vivo* conditions.

### 4.3 Insight From the Structurally Informed Model

The phenomenological computational models typically provide good capture of the macroscopic mechanical behavior; however, they lack detailed information on the structural and material properties of the myocardium constituents (Avazmohammadi et al., 2019a). Instead, the structural constitutive model, such as the one used in this study, is formulated to capture the underlying microstructural mechanisms for the macroscopic behavior of the tissue. From our results, the LV tended to have a larger transition strain than the RV, which suggests that the LV recruit collagen later than the RV. Furthermore, the LV tended to have a larger $k_{1}^{m}$ value (myofiber stiffness) and a larger $k_{1}^{c}$ value (collagen stiffness) than those of the RV, suggesting a stiffer fiber material property. This finding is similar to the recent report of Kakaletsis et al. (2021). The cause for stiffer collagen fibers in the LV than the RV awaits further investigation. The ventricular differences in the diastolic function and the adaptation to mechanical loading conditions depend on microstructural characteristics like myofiber and collagen stiffness. The structurally informed model here can help to highlight the myofiber and collagen contributions to that of organ-level remodeling, which will in turn help to develop targeted therapies that prevent or reverse maladaptive remodeling (Avazmohammadi et al., 2019c).

### 4.4 Correlations Between Collagen Content and Ventricle Elasticity

In this study, we also found novel correlations between collagen and ventricular biomechanics. Firstly, we observed a trend of higher collagen content including type I collagen in the RV compared to the LV. This is consistent with the prior report of collagen content in human LV and RV determined from hydroxyproline assays (Miles et al., 2020). Second, although type I collagen is the most abundant type of collagen in ventricles (LeBar and Wang, 2021), the type III collagen content was significantly correlated with the *M*_*L*_ in all ventricles and at low strains (Figure 7B, *p* < 0.05). This indicates that the longitudinal elasticity partly stemmed from type III collagen recruitment. It is known that type III collagen is mesh-like in structure and more compliant than fibrillar type I collagen (Silver et al., 2002), but how these fibers are recruited during the nonlinear deformation is unclear. Our data suggest that the type III collagen may play an equally important role as myofiber in low-strain tissue mechanics, which is key to ventricular diastolic function (Jang et al., 2017). It is also possible that type III collagen is recruited earlier than type I collagen and presents a similar role as elastin in vascular tissues. Lastly, the total collagen content was significantly correlated with the circumferential *M/ε* in both ventricles and at high strains (Figure 7C, *p* < 0.05). The strong influence of collagen fibers in high-strain elasticity is likely the outcome of more fully recruited collagen at larger deformation.

### 4.5 Limitations

Several limitations were present in the study. Our samples were from female ovine. Sex differences have been found in ventricular function in both physiological and pathological conditions (Lahm et al., 2018), but its effect on the myocardium mechanical property has not been reported. Next, the middle portion of the ventricles were tested to fulfill the plane stress requirement in biaxial test. This was viewed as standard for biaxial tests of myocardium (Witzenburg et al., 2012). But it resulted in an incomplete characterization of tissue mechanics and transmural fiber orientation, especially in the LV wall. Such limitation is not rare for *ex vivo* mechanical measurement of myocardium from large animals or human patients due to a large tissue size. Prior studies typically sectioned the LV into two or three layers (Sommer et al., 2015; Javani et al., 2016; Fatemifar et al., 2018) or at different anatomical regions (Javani et al., 2016; Ahmad et al., 2018), and then performed the biaxial tests. However, the entire ventricle’s mechanical behavior is “interrupted” by sectioning. Therefore, the full description of mechanical properties of the LV (or hypertrophied RV) would require the development of *in vivo* computational modeling using intact, complete structural information of the patient. Third, the samples were sectioned in cruciform shape and mounted by clamps in our biaxial tests, similar to a prior rat RV study (Witzenburg et al., 2012). We chose this method based on a prior examination of our in-house biaxial system on brain tissues (Labus and Puttlitz, 2016). The impact of sample shape and mounting method on biaxial tests has been explored by Sun et al. (2005). We acknowledge that our methodology is different than other studies with square samples and sutures mounting, but an examination of the strain data indicates relatively homogenous deformation in the center region of the tissue. Thus, we expect that the discrepancy induced by this methodology should be minimal. Furthermore, we tested the samples at room temperature with a regular spray of PBS rather than immerse the tissue in a relaxant solution at body temperature. We investigated the effects of these testing conditions on the passive mechanical properties by using extra ventricles. We compared the stress-strain curves and observed no significant changes of the mechanical behavior between these two conditions.

Lastly, myocardium is a nonlinear, orthotropic, and viscoelastic material. In order to fully characterize the mechanical property, the combination of shear and biaxial tests and the inclusion of viscoelasticity measurement are recommended (Holzapfel and Ogden, 2009; Sommer et al., 2015). Nevertheless, the *ex vivo* planar biaxial test is still widely performed in cardiac mechanical testing (Voorhees and Han, 2015), and it provides an initial examination of passive mechanical properties that are independent of physiological conditions such as *in vivo* pressure and volume, heart rate, sympathetic nervous stimulation, etc. The study of biaxial planar mechanics is critical to understand RV diastolic function as the deformation replicates the physiological motion (Pettersen et al., 2007; Haddad et al., 2008; Taverne et al., 2020). The relatively simple testing protocol minimizes the testing time to ensure tissue viability (Valdez-Jasso et al., 2012; Avazmohammadi et al., 2018). Therefore, this method remains common to characterize myocardium passive properties (Ghaemi et al., 2009; Hill et al., 2014; Javani et al., 2016; Park et al., 2016; Avazmohammadi et al., 2017b; Ahmad et al., 2018; Fatemifar et al., 2018; Vélez-Rendón et al., 2019).

## 5 Conclusion

In this study, we examined the biomechanical differences between healthy LVs and RVs in adult ovine. We observed differences in the anisotropic behavior between the LV and RV, with the LV being stiffer in the longitudinal (OT) direction and the RV being stiffer in the circumferential (cross-OT) direction. Interventricular comparison showed that the RV was more compliant than the LV in the longitudinal direction and was stiffer than the LV in the circumferential direction, which suggests different impacts of passive mechanics of these ventricles on the blood filling during diastole. These anisotropic properties were captured by the zero-load elastic moduli as well as the anisotropic parameter *K* derived from the four-parameter Fung type model. Results from the structurally informed model imply stiffer collagen fibers in the LV than the RV, which awaits further investigation. Moreover, type III collagen content was correlated with the low-strain elastic moduli in the longitudinal direction in both ventricles. Our findings provide significant insights for guiding cardiac tissue engineering and regenerative studies and call for the development of RV-specific therapy based on its unique biomechanics. In addition, our results can serve as a benchmark for image-based inverse modeling technologies to non-invasively estimate myocardial properties in various types of heart failure patients.

## Data Availability

The original contributions presented in the study are included in the article/Supplementary Material, further inquiries can be directed to the corresponding author.
